# Remarks on the Remedial Virtues of Cannabis Indica, or Indian Hemp

**Published:** 1855-12

**Authors:** George S. D. Anderson

**Affiliations:** Rapides, La.


### ART. IV.—REMARKS ON THE REMEDIAL VIRTUES OF CANNABIS INDICA, OR INDIAN HEMP.

BY GEORGE S. D. ANDERSON, M. D., OF RAPIDES, LA.

THOUGH the Greek physicians were unacquainted with the extraordinary narcotic properties of the Indian hemp, it has been known in the East Indies and other parts of the Eastern Continent as an intoxicating and medicinal agent from a very early period. Christison says it was known to the Hindoos so early as the year 600, and that the Persians became acquainted with it not long afterwards; at a subsequent period its virtues became known to the Arabians, by whom, as well as by the Persians, it was used medicinally as a sedative and anodyne. According to Dunglison, its properties as a narcotic have long been known to the people of Southern Africa, South America, Turkey, Egypt, Asia Minor, and India; it has also been known to the Maylays, Burmese, and Siamese, being used both as an intoxicating and therapeutic agent.

Notwithstanding the fact that Indian hemp has for more than twelve centuries been known in India and other portions of the Eastern Continent as a powerful narcotic, and has also been frequently noticed by travelers and others who have given accounts of it, it does not seem to have been known in Western Europe either as an intoxicating, toxicological, or therapeutic agent, till Dr. O'Shaughnessy, of Calcutta, published an account of it in 1839. To him, therefore, is due the credit of first giving a precise account of its medicinal properties.

Since the publication of Dr. O'Shaughnessy's account of the virtues of the Indian hemp, it has been used as a medicine by many respectable practitioners both in Europe and America. The accounts, however, given of its effects on the economy by different experimenters, are somewhat contradictory. But experience abundantly proves it to be a most powerful narcotic, capable of answering important indications in the treatment of disease. Like all other potent medicines it should be administered with caution and judgment, as an over-dose produces alarming symptoms; so much so, that one who has witnessed the effects of an over-dose might be induced to abandon it altogether as a medical agent. But however powerful as a narcotic it may be, and however alarming the symptoms of an over-dose, we cannot subscribe to the opinion, that because it is one of our most potent medicines, capable of destroying life in not a very large dose, its use should be abandoned. As well might we condemn and abandon the use of strychnine, morphine, veratria, tartar emetic, and many other valuable articles of the Materia Medica; and so the surgeon might abandon the knife, because in the hands of empirics and others unskilled in its use, it has done harm. We hold, therefore, that the mere fact of Indian hemp being a very powerful medicine, capable of doing harm when incautiously or unskillfully administered, is not ground sufficient for rejecting it as a therapeutic agent, than which none, perhaps, is more certain as an anodyne, relieving pain almost like a charm.

We have used the Indian hemp in six instances—twice in the same individual—and we propose to lay our experience before the profession for the purpose of directing the attention of physicians more particularly to it.

The first case was that of a gentleman about twenty-five years of age. He had been the subject of pleurodynia for several years, having suffered a great deal with it while at college. The suffering experienced during an attack was intense, and, in some instances, threatened almost instant death. He was generally attacked in the night, and most commonly after having taken butter-milk for his dinner or supper. We believe that other articles of diet also brought it on, or seemed to act as an exciting cause. Before consulting me he had been in the habit of taking large doses of morphine and of chloroform, internally, in the same attack. These medicines generally afforded relief, but sometimes many hours have elapsed before complete relief was obtained. I considered this a case well suited to the Cannabis Indica; accordingly I procured an excellent article of the extract for the purpose of making a trial of it the first opportunity that presented itself. The patient had an attack, as usual in the night, and morphine and chloroform had both been given before I saw him, though I do not now recollect how much of either. When I saw him he was suffering greatly: I immediately gave him a grain of the extract of Indian hemp. In a short time he expressed himself much better and became sleepy. About half an hour after the first dose, I repeated the medicine, giving him another grain, when he went to sleep and slept well the balance of the night, getting up quite well next morning, without any unpleasant effects from the medicine, such as head-ache, stupor, etc.

On a subsequent occasion I saw the patient at the beginning of an attack, before he had taken any medicine. I immediately gave him thirty drops of a tincture prepared from the above extract, of the strength of one grain of the extract to sixteen minims of dilute alcohol. This dose did not afford a like relief, so I in a short time repeated it, when he became easy and again went to sleep and slept well all night. He experienced some head symptoms next morning, as dullness, etc.

The two doses in this last case were about equal in amount to two grains of the extract.

The third case was that of an infant in its fourth month with a spasmodic colic. It had taken Dewees' carminatives and tinct. opii. camph. before I saw it. I have no recollection of the quantity of either of these given, but whatever the doses may have been, (small, though, I suppose, as it was the first child, and its fond mother was timid,) relief had not followed its administration. When I saw it I gave it one drop of the above tincture with three drops of tinct. opii. camph., after which it soon composed itself to sleep, and awoke quite refreshed and free from pain.

The fourth case was one of fibro-bronchitis, in a gentleman about forty-five years of age. He had been rather suddenly attacked on the evening of the 23d of December, 1854, when I was immediately sent for, and found him breathing very hurriedly—almost panting. I bled him from the arm immediately, ordered a warm bath, and gave him tincture of Indian hemp, bled him again in about half an hour, and repeated the bath and tincture of Indian hemp, with tincture of lobelia, fifteen drops. Before he left the bathing-tub he became almost deathly sick, had an operation from the bowels immediately afterwards, and took his bed. The sickness continued for some time, but the difficulty of breathing rapidly passed away, and in the course of about three hours he became sleepy, and rested tolerably well the remainder of the night. He recovered under the use of the compound syrup of squills, etc., expectorating tube-like matter in the course of three or four days. This matter was rather ash-colored and in quills about the size of a common writing quill, and was very tough and stringy.

The fifth case was that of a lady in the eighty-seventh year of her age. She had neuralgia of the right side of the head, affecting particularly the right eye. It was paroxysmal, returning every morning between eight and nine o'clock. Quinine and morphine had been used in this case, but had not relieved the patient of the pain. She suffered from a return of each paroxysm. I therefore determined to try the Indian hemp in this case. Accordingly, on the morning of the 20th of Feb., 1855, I repaired to her house about six o'clock, A. M., and found my patient had just risen from bed, and entirely free from pain. I gave her about one-fourth of a grain of morphine and five drops of the tincture of hemp. About an hour afterwards I repeated the tincture. She had now taken two doses or about ten drops, and experienced no effects from it. About an hour and a half after this she complained of a return of her neuralgia, when I gave her about the eighth of a grain of morphine, with six drops of the tincture of Indian hemp. She soon complained of being too warm, and began to perspire freely. The doors and windows were thrown open, and she was fanned. She now began to complain of sickness at the stomach; the perspiration increased; the pulse became smaller and smaller at the wrist till finally it could not be felt; the superficial veins of the hands and arms became distended to their utmost, refusing to contract to force the blood downwards to the heart; and her eyes assumed a deathly position and aspect. Such was the alarming spectacle before me. I at once commenced giving pure cologne (the most powerful stimulant in the house), before she had approached the worst. She took several teaspoonsful before she ceased to swallow, and then by rubbing with warm cloths, as well perhaps as by the noise made by the servants, she ere long showed signs of returning animation and recovered. She had no return of the neuralgia, nor has she had a return of it since till within the last few days, (13th of December, 1855.)

The peculiar effects of the medicine were manifested in this case; for after recovery from the state of narcotism it induced, she said she felt as though she had no head, nor could she tell anything about the feeling in her hands, but an indescribable feeling pervaded her entire system.

This case had the effect of inducing me to abandon for a time the use of the Cannabis Indica. I have used it but once since; that was in the case of a negro woman, about forty years of age, with dysentery. I gave her several doses of it, always with the effect of affording relief, but as she had other diseases that were chronic it became necessary to abandon its use, and I have not used it since. This occurred in April, 1855.

I offer these cases without comment. I did not, it will be seen, confine myself to any particular class of diseases; nor can my experiments be considered at all conclusive as to what all the effects of the medicine may be when administered for medicinal or other purposes. Enough was shown to convince me of the extraordinary powers of the medicine, and I have every reason to believe that it will, in time, become one of our most valued and esteemed medicinal agents. That it should be cautiously used is evinced by the last case but one enumerated.

*December* 17, 1855.

